# Draft Genome Sequence of the Lytic *Salmonella* Phage OSY-STA, Which Infects Multiple *Salmonella* Serovars

**DOI:** 10.1128/MRA.00868-20

**Published:** 2020-08-27

**Authors:** Yue Yi, Ahmed G. Abdelhamid, Yumin Xu, Ahmed E. Yousef

**Affiliations:** a Department of Food Science and Technology, The Ohio State University, Columbus, Ohio, USA; b Department of Microbiology, The Ohio State University, Columbus, Ohio, USA; DOE Joint Genome Institute

## Abstract

Bacteriophage OSY-STA is a new anti-*Salmonella* phage that was isolated from a chicken farm in Ohio. It is a promising candidate for food safety applications, considering its efficiency in infecting several Salmonella enterica serovars. The current work presents its genomic characteristics. S*almonella* phage OSY-STA has a 111,039-bp genome and 166 open reading frames.

## ANNOUNCEMENT

The use of bacteriophages in controlling foodborne pathogens has been accepted as a green technology that enhances the safety of food. Bacteriophages can be used to prevent pathogen colonization in livestock (1) and to serve as natural food preservatives (2). In a previous study, a *Salmonella* phage designated OSY-STA was isolated from the organs of chickens slaughtered at the Poultry Research Center at The Ohio State University (Wooster, OH, USA) (3). The phage was capable of infecting many *Salmonella* serovars and was effective against *Salmonella* strains inoculated in liquid egg (3). The current study reports the genome sequence of *Salmonella* phage OSY-STA, with the goal of providing insights into its biological characteristics.

Phage OSY-STA was propagated in a culture of Salmonella enterica serovar Typhimurium and separated as described previously (4). The phage genomic DNA (gDNA) was extracted from a 3-ml pure phage suspension (10^9^ PFU/ml) using a phage DNA isolation kit (Norgen Biotek Corp., Thorold, Ontario, Canada), following the manufacturer’s protocol. The gDNA quantification and purity check were performed spectrophotometrically (NanoVue Plus; Biochrom USA, Holliston, MA). The extracted gDNA was used for the preparation of DNA libraries using a library preparation kit (Nextera XT; Illumina, Madison, WI) according to the manufacturer’s instructions. Library quantification and sizing were performed using a fluorimeter (Qubit; Invitrogen, Waltham, MA) and an automated gel electrophoresis system (TapeStation 2200; Agilent Technologies, Santa Clara, CA), respectively. Pooled libraries were sequenced using a DNA sequencer (Illumina MiniSeq platform; Sequencing Centre Company, Fort Collins, CO). The quality of paired-end raw reads (150-bp size) was checked using FastQC v0.11.9 software (5). This was followed by trimming the adapters and low-quality N bases and removing duplicate reads using Geneious Assembler v9.0.5 software (6). The cleaned raw reads (1,295,764 reads) were used for *de novo* genome assembly using the Geneious Assembler; this resulted in one contig, and the read coverage for the final assembled genome was 1,235×. The assembled genome was annotated using a bioinformatic server (MyRast [https://rast.nmpdr.org]) to determine the open reading frames (ORFs) for genes encoding proteins. The tRNAScanSE search tool (http://lowelab.ucsc.edu/tRNAscan-SE/index.html) was used to search for genes encoding tRNAs. Functional annotation of the ORFs obtained was performed using BLASTp (https://blast.ncbi.nlm.nih.gov/Blast.cgi?PAGE=Proteins) with the NCBI nonredundant protein database. All software and bioinformatic Web servers were used with their default settings.

OSY-STA is a double-stranded DNA phage. The genome is linear, as determined with the PhageTerm tool (7), and contains 111,039 bp with a GC content of 40.0%. A total of 166 ORFs were identified, with only 42 ORFs determined to encode functional proteins. The phage OSY-STA genome encoded a holin and an endolysin, whereas lysogeny-associated genes were absent. The existence of both holin- and endolysin-encoding regions suggests that the phage utilizes a holin-endolysin system to disrupt host cell membranes (8). A total of 28 tRNAs were identified in the phage genome. No toxins such as the ADP-ribosylating toxins (9) or virulence-related proteins (10), which are specific to S. enterica, were identified in the OSY-STA genome. Average nucleotide identity analysis, performed with the JSpeciesWS tool (http://jspecies.ribohost.com/jspeciesws), revealed similarity of OSY-STA to two T5-like phages, namely, *Salmonella* phages 3-29 (97.2%) and BSP22A (96.88%).

### Data availability.

The genome sequence of *Salmonella* phage OSY-STA was deposited in GenBank and assigned the accession number NC_048808. The associated BioSample, BioProject, and SRA accession numbers are SAMN12286159, PRJNA554893, and SRS5109868, respectively.
